# Aging and Burnout for Nurses in an Acute Care Setting: The First Wave of COVID-19

**DOI:** 10.3390/ijerph20085565

**Published:** 2023-04-18

**Authors:** Margaret E. Beier, Mona Cockerham, Sandy Branson, Lisa Boss

**Affiliations:** 1 Department of Psychological Sciences, Rice University, Houston, TX 77005, USA; 2 School of Nursing, Sam Houston State University, The Woodlands, TX 77380, USA; 3 Cizik School of Nursing, The University of Texas Health Science Center, UT-Health, Houston, TX 77355, USA; 4 School of Nursing, Tarleton State University, Fort Worth, TX 76402, USA

**Keywords:** aging, COVID, burnout, coping, nurse

## Abstract

We examined the relationship between age, coping, and burnout during the peak of the COVID-19 pandemic with nurses in Texas (N = 376). Nurses were recruited through a professional association and snowball sampling methodology for the cross-sectional survey study. Framed in lifespan development theories, we expected that nurse age and experience would be positively correlated with positive coping strategies (e.g., getting emotional support from others) and negatively correlated with negative coping strategies (e.g., drinking and drug use). We also expected age to be negatively related to the emotional exhaustion and depersonalization facets of burnout and positively related to the personal accomplishment facet of burnout. Findings were largely supported in that age was positively associated with positive coping and personal accomplishment and age and experience were negatively correlated with negative coping and depersonalization. Age was not, however, associated with emotional exhaustion. Mediation models further suggest that coping explains some of the effect of age on burnout. A theoretical extension of lifespan development models into an extreme environment and practical implications for coping in these environments are discussed.

## 1. Introduction

Under normal conditions, healthcare workers are exposed to complex, stressful, and emotionally draining situations that increase the risk of professional burnout [1]. Burnout is a psychological condition characterized by feelings of helplessness, cynicism, and reduced efficacy [2]. Among healthcare workers, nurses have historically been shown to have the highest prevalence of burnout, perhaps due to heavy workload, limited resources, and the emotional labor involved with engaging with patients who are unwell, patients’ families, and doctors [1]. Under extraordinary conditions, such as those imposed by the COVID-19 pandemic, one would expect burnout to increase significantly for all healthcare workers, and indeed this was the case [3]. Moreover, as might be expected given their levels of burnout under normal conditions, nurses were especially negatively affected by the pandemic [4]. For instance, the American Nurses Foundation and the American Nurses Association launched the Pulse on the Nation’s Nurses survey series at the start of the COVID-19 pandemic and have been administering the survey every year since. Results from the second year of the COVID-19 pandemic, administered in January 2022, showed that over 70% of the over 11,000 nurses surveyed reported feeling stressed in the past 14 days and 65% reported feeling exhausted during that same time period; these values actually showed increases compared to the first year of the pandemic when 43% reported feeling stressed and 51% reported being exhausted [5]. Because conditions like those imposed by COVID-19 are constantly changing and have become long-term challenges in healthcare, understanding the determinants of burnout in nursing under such extremes can help in managing nurse burnout in the future.

Research suggests that older workers tend to experience less burnout relative to younger workers [6,7]. The negative relationship between age and burnout has also been shown in research on nurses during the COVID-19 pandemic [8]. Given relative declines in many abilities—e.g., memory abilities, abilities related to solving novel problems [9]—the buffering effect of age on burnout is perhaps surprising. However, lifespan development theories point to the importance of experiences that build problem-solving skills with age, as well as age-related increases in the capacity to regulate emotions and manage stress [10,11,12]. Thus, older nurses likely benefit from their experience—dealing with stressors both at work and at home—to solve problems during times of extreme crises. Little is known, however, about the behaviors and thought processes that might explain the negative relationship between age and burnout in nurses. In this study, we examine coping behaviors as explanatory mechanisms for the negative relationship between age and burnout.

The purpose of the current study was to examine the relationship between age and burnout in a cross-sectional study with a convenience sample of nurses working in Texas during the height of the COVID-19 pandemic. Further, we examined the behaviors, in terms of positive and negative coping mechanisms, that can help explain the relationship between age and burnout. Framed within lifespan development theories, we expected that age would be negatively associated with burnout, given that life and work experiences provide nurses with the knowledge and emotion regulation skills that would mitigate the effects of the pandemic on burnout. We also expected that coping behaviors would help explain the relationship between age and burnout, and that positive coping behaviors would be positively correlated with age and negative coping behaviors negatively related with age. Our study contributes to the lifespan development literature by providing evidence of the benefits of age and experience on job burnout. Moreover, we identify the coping behaviors that explain this relationship. Examining nurse burnout in such an extreme environment (e.g., during COVID-19) provides insight into how nurses can handle these types of environments to keep burnout at bay.

### 1.1. Burnout

Burnout is defined as a “syndrome of emotional exhaustion and cynicism” [2] frequent among people working in helping and client-facing roles. Although there are an array of theoretical formulations of burnout, accompanied by their own approaches to measurement, the most prevalent is the Maslach and Jackson, which identifies facets of burnout including: (a) emotional exhaustion, reflecting depletion of psychological resources for engaging with those they help, (b) cynical attitudes towards patients, also called depersonalization, and (c) a reduction in feelings of efficacy and mastery in work roles; that is, a sense of lacking personal accomplishment [13]. Many formulations of organizational burnout conceptualize it as the opposite of work engagement, which is defined as employing one’s cognitive, physical, and emotional selves at work [14]. The expectation is that people who are engaged will be relatively low on emotional exhaustion and depersonalization and relatively high on personal accomplishment, and people experiencing burnout will display the opposite pattern of relationships [15]. Still, others have argued that the emotional exhaustion facet is the central component of burnout and tends to ignore the depersonalization and personal accomplishment facets as symptoms of burnout [13]. In the current study, we examine all three facets of burnout [2]. That is, we assume that nurses experiencing burnout are likely to feel psychologically and emotionally drained and ineffective in their roles, and they may become callous towards those they are focused on helping the most, their patients. Job demands (e.g., aspects of jobs that require sustained physical, mental, or emotional effort) and job resources (e.g., social, physical, psychological, or organizational aspects that support workers in doing their jobs) are important antecedents of burnout. Theoretically then, burnout happens in situations where resources, such as social support, organizational support, autonomy in decision making, are lacking [16] and when job demands are high. Research suggests, however, that job demands are more important than resources for predicting burnout [15]. Although we do not examine job demands and resources per se, the current study took place during the height of the COVID-19 pandemic in Texas, where job demands for all healthcare professionals were extensive [17].

Nursing is a highly stressful profession often triggered by prolonged stress or exposure to stressors, and these stressors were exacerbated by the outbreak of the COVID-19 pandemic. For example, frontline healthcare workers working during the COVID-19 pandemic were exposed to threats to physical safety from infection, insufficient resources, continuous changes in roles and responsibilities, high patient acuity and mortality rates, long shifts, inadequate rest, and inadequate personal protective of equipment [18]. Each of these factors likely increase perceived job demands and reduced perceived and real resources in ways that exacerbated burnout for healthcare workers. One study in China reported a high level of fear and burnout during the early outbreak of the COVID-19 pandemic, with 40–60% of healthcare workers reporting that they were experiencing all attributes of burnout (i.e., emotional exhaustion, depersonalization, and a lack of personal accomplishment) [19]. This finding has been replicated for healthcare workers in other parts of the world [20]. During the early days of the pandemic, many nurses worked long hours, fearful of spreading the disease to family and thus they experienced isolation from the public and even within their own organization for fear of exposure and even death [20]. Because burnout leads to physical and emotional symptoms that contribute to withdrawal from work, including sickness absence and turnover [21], it is vitally important to understand the antecedents of burnout in nursing, particularly in the context of projected nursing shortages [22].

### 1.2. Aging and Lifespan Development

According to the lifespan development perspective [10], aging is an ongoing process of gains and losses in different domains, such as cognition, motivation, and self-regulation. Age-related losses are perhaps the best understood and documented, including declines in some cognitive abilities (e.g., memory and reasoning) [9]. These losses are expected to begin as early as late adolescence and early adulthood [9], although cross sectional research tends to show these declines starting earlier than longitudinal studies do [23], and there are certainly individual differences in rates of decline [24]. Nonetheless, age-related declines in abilities tend to be apparent by early adulthood (i.e., age 30 or so) [9].

Although researchers have traditionally focused almost exclusively on age-related declines, lifespan development theories also highlight growth that accompanies normal healthy aging [10]. Specifically, these models posit that people tend to accumulate knowledge and skills through their experiences in educational, work, and home environments and that they bring these skills to bear to successfully manage themselves and others on a daily basis [11]. Growth in knowledge includes technical knowledge (e.g., knowledge about checking vital signs) and the ability to deploy emotion regulation strategies to buffer the psychological stress associated with the complex problems one encounters on a daily basis [11]. In support of this idea, research suggests that older adults are more likely to focus on positive, as opposed to negative, stimuli (i.e., the positivity effect) more than younger adults do [25]. Research also suggests that—although relative standing on personality traits stays somewhat constant throughout the lifespan—mean levels in traits related to emotional stability tend to increase throughout the lifespan [26]. In sum, despite the well-document declines in some abilities with age, people tend to accumulate the knowledge and skills that permit them to operate in highly complex environments, including those that ward off negative emotional effects associated with working in stressful work environments. That is, people become more emotionally stable, they develop a tendency to focus on the positive rather than the negative, and they become better able to manage their emotions as they age.

Although lifespan development models focus on age as an index of development, age is confounded with experience in these models. That is, it is not chronological age per se that is important for developing the knowledge and skills to handle complex environments, but the experiences acquired through aging that contribute to this development [10]. As people age, for instance, they will have experiences in their daily lives, such as dealing with difficult family members, caring for children and/or elderly parents, that will support the development of skills related to handling complex relationships at work. In the context of nursing, job experience dealing with complex situations and relationships with patients, families, doctors, and other nurses will also lead to the development of knowledge and skills necessary to deal with difficult and stressful environments [27]. In the current study, we will examine the relationship between age in concert with job experience.

Research on work-related burnout tends to support ideas put forth by the lifespan development framework. A meta-analysis of 36 published effects across occupations found a small, but significant, negative relationship between age and burnout and reported a similarly-sized effect for the relationship between job experience and burnout [6]. However, research also suggests that the relationship between age and job burnout is not straightforward. Reh et al. examined the relationship between age and job satisfaction and affect (positive and negative) as moderated by emotional job demands [12]. Emotional job demands are defined as those aspects of a job that require employees to manage and control their own emotions or to induce emotional responses in others [28]. These types of job demands are particularly prevalent in jobs, such as nursing, that have high client-facing components. As expected from a lifespan development perspective, Reh et al. hypothesized that experience in jobs with high emotional job demands would buffer the effect of these demands on job satisfaction/positive affect [12]. What they found, however, was that when older workers experienced jobs that were high in emotional demands, they were likely to experience *less* positive affect/job satisfaction compared to younger workers. The authors posit that combined with declines in physical and cognitive abilities with age, emotional job demands served to overload workers, reducing their job satisfaction and positive affect. It is notable that the mean age of the sample used in the Reh et al. study was 53 years old at the final measurement occasion, an age at which cognitive and physical abilities are indeed likely to be in decline [29]. The mean age of nurses in the current study is just over 33 years old with a standard deviation of about 8 years. Although many of the nurses in the current study may have been experiencing age-related declines in abilities, these declines may not have been significant enough to overwhelm them when added to their stressful and complex daily work during the height of the COVID-19 pandemic. Notably, the age of nurses in our sample is markedly younger than the average age of nurses in the U.S., which is 52 years old, but is closer to the age of nurses at the bedside (i.e., the majority being between 25 and 40 years old) [30].

In sum, the lifespan development framework provides the rationale for why age and job experience would be negatively related to burnout. Specifically, as people age, they develop the knowledge and skills associated with emotions and emotion regulation such that they are better equipped, on average, to deal with negative and stressful events. Thus, we hypothesize the following:

**Hypothesis** **1.** *Nurse age (H1a) and nurse job experience (H1b) will be negatively related to burnout. Specifically, they will be negatively associated with emotional exhaustion and depersonalization facets of burnout and positively related to the personal accomplishment facets of burnout.*

We also examined the behaviors—specifically related to coping—that can help explain why age/job experience are negatively related to burnout.

### 1.3. Coping

Coping is defined as “the process of managing demands (external or internal) that are appraised as taxing or exceeding the resources of the person” [31], and it occurs when a person struggles with, but manages, stress [32]. Coping strategies are fundamental to a person’s socio-emotional functioning and influence overall health and well-being. Everyday encounters of stress (daily hassles) as well as those resulting in tremendous stress, such as those that might be encountered in a healthcare setting during the height of the COVID-19 pandemic, elicit a person’s use of coping strategies [33]. The use of these strategies is influenced by the appraisal of the options available to the person, and coping strategies can vary in their effectiveness [34].

There is an array of theoretical formulations associated with coping. For instance, researchers have examined problem-based coping, which focuses on changing one’s environment to solve the problem and emotion-based coping, which focuses on expressing, managing, or changing one’s emotional response to a stressor. A meta-analysis on the relationship between problem- and emotion-based coping and burnout shows that problem-based coping is negatively associated with burnout, while emotion-based coping can exacerbate burnout (Shin et al., 2013). A study of nurse age, problem- and emotion-based coping, and burnout further suggest that problem-based coping is a more productive strategy in mitigating the effects of burnout, while emotion-based coping strategies may be most problematic for older nurses [35].

Rather than focusing on problem- or emotion-based coping, many researchers using one of the most widely-known measures of coping, the COPE [34] or the Brief-COPE [36], often report findings in the context of approach- or avoidance-oriented strategies. Approach-oriented coping strategies are associated with a lessened burden of stress and are considered positive. Specific examples of positive coping include planning, positive reframing, social support, human–animal interaction, acceptance, and the use of meditation, exercise, and/or hobbies. Avoidance coping strategies are associated with an increased burden of stress and are considered negative. Specific examples of avoidance coping include denial, disengagement, substance abuse, self-blame, behavioral disengagement, and venting [37]. Further, approach coping strategies are associated with optimal health outcomes, including less anxiety, depression, stress, and better overall well-being, engagement, quality of relationships, and accomplishment. By contrast, adverse health outcomes are commonly associated with avoidance coping strategies and include post-traumatic stress disorder, depression, and anxiety [38,39].

Healthcare workers suffered a high prevalence of anxiety, burnout, depression, post-traumatic stress disorder (PTSD), psychological distress, and overall deterioration of mental health and well-being during the COVID-19 pandemic [40,41]. Accordingly, mounting evidence shows the choice of coping strategy may be important for influencing health outcomes. For instance, there is strong evidence that approach coping strategies were associated with better mental health outcomes in healthcare workers during the COVID-19 pandemic [42,43,44,45,46,47,48]. Likewise, the use of approach coping strategies was associated with better resilience for medical professionals sent to Wuhan China during the start of the COVID-19 pandemic [49]; whereas avoidance coping strategies were associated with occupational burnout [20]. There are also mixed findings regarding coping during the pandemic. In two studies with healthcare workers, use of a approach and problem-focused coping strategy was a protective factor for anxiety and depression [42,50], while another author reported that use of an approach and problem-focused coping strategy was associated with higher psychological distress in nurses [44]. Additionally, the use of religious practices, considered a positive coping strategy, was not significant in reducing stress during the pandemic [42,46]. However, healthcare workers with higher levels of hope and optimism associated with spirituality showed less anxiety during the pandemic [46].

The current study will further examine the effects of coping on burnout. Despite mixed findings, we expect that positive coping will lead to less burnout in the nurse sample studied here; and that negative coping will lead to more burnout and thus hypothesize the following:

**Hypothesis** **2.** *Positive coping will be positively related to personal accomplishment (2a), but will be negatively related to emotional exhaustion (2b) and depersonalization (2c).*

**Hypothesis** **3.** *Negative coping will be negatively related to personal accomplishment (3a), but will be positively related to emotional exhaustion (3b) and depersonalization (3c).*

There is limited research examining the relationship between coping and age, and furthermore, the results of this research show mixed results. In general samples, Folkman et al. found that the use of problem-focused coping decreased with age, although emotion-based coping increased [51], while Aldwin reported that problem-focused coping is maintained with age [52]. Another study found that older participants reported using few coping strategies and did not find them to be helpful [53], and other researchers reported no difference in coping strategy efficacy, even though finding that older adults reported using fewer coping strategies [54,55].

In recent studies examining age and coping with healthcare workers during COVID-19, preliminary findings are also scant, but largely indicate that younger age predicts less positive, adaptive coping and more perceived stress [43]. Bozdağ and Ergün also found that the age of healthcare workers was positively correlated with using positive coping strategies and that these led to better psychological resilience [56]. Similarly, Maiorano et al. reported that older healthcare workers used more positive coping strategies during COVID-19 that reduced negative thoughts and emotions, so that they were less influenced by negative, intrusive thoughts [45]. Aligned with these preliminary findings, we expected that age will be positively associated with positive coping strategies and negatively associated with negative ones.

**Hypothesis** **4.** *Nurse age (H4a) and nurse job experience (H4b) will be positively correlated with approach oriented/positive coping strategies.*

**Hypothesis** **5.** *Nurse age (H5a) and nurse job experience (H5b) be negatively correlated with avoidance oriented/negative coping strategies.*

Although research examining age, coping, and burnout is limited, there has been some research that has examined the moderating effect of coping on the relationship between age and burnout [35]. This study found that emotional coping moderated the relationship between age and burnout, such that older adults who engaged in emotion-based coping strategies experienced more burnout than older adults who did not engage in these strategies. Our study is based on the lifespan development perspective, however, and as such we examine coping as a mediator between age and burnout. That is, we expect that age and job experience will lead to better coping through the development of more effective coping strategies over one’s lifespan. Further, we expect that the relationship between age and burnout would be—in part—explained by the coping strategies that a person learns through their life and job experiences [10,11]. In sum, we posit that people not only become more emotionally stable with age (and able to deal with adversity), but they also develop coping strategies that help them deal with job demands, which in turn mitigate burnout. As such, we expected that coping will mediate the relationship between age and burnout, and thus hypothesize the following:

**Hypothesis** **6.** *(H6) Coping strategies will mediate the relationship between age/job experience and burnout.*

## 2. Methods

### 2.1. Participants and Procedures

The current study was part of a larger project examining the experiences of nurses during the COVID-19 pandemic. One study associated with this project has been previously published [57]. The previously published study examined PTSD in nurses working directly in COVID-19 units. In the current study, we were interested in burnout experienced by nurses working during the COVID-19 pandemic. Burnout is a more typical response to stressful work situations compared to PTSD, and as such, we used a broader sample of nurses who worked during the first wave of COVID-19 outbreak from May–July 2020 in Texas. There are no variables in common between the published study and the current study.

This study was approved by Institutional Review Boards at the universities where the researchers are based. All subjects gave their informed consent for inclusion before they participated in the study and were informed of the risks and benefits, voluntariness, and confidentiality of collected data in the consent form. We recruited nurses through the Texas Nurses Association. Specifically, we sent nurses emails with a link to a Qualtrics survey. Nurses were also recruited via snowball sampling methodology. Because the goal of the original project was to understand the general experiences of nurses during the pandemic, and not nurse age per se, we did not use a targeted sampling approach to ensure equal distribution of nurses across age groups and experience levels. Moreover, given that the healthcare environment was rather chaotic at the time of our surveying, we had no way to know the age range of nurses working during that time. As such, we decided to take the opportunity to survey all nurses who wanted to complete our survey.

Nurses completed a 165-item survey that included demographic data (age, gender, job title, hospital city/state location, years of experience in the current hospital, education, and whether they worked on a specialty COVID unit or not); they also completed questionnaires associated with a larger project (PTSD symptomology, adaptability, and perceived organizational support) as well as the assessments described below [57].

We collected data from May through July, 2020, considered the “first wave” of the COVID-19 pandemic during an increase in COVID-related cases and deaths in Texas (i.e., there were about 90,000 new cases of COVID in June 2020 and over 250,000 new cases in July 2020; new deaths rose from just over 1000 in June 2020 to over 6500 in July 2020 [17]). Participating nurses were given a $5 gift card upon completion of the survey and 945 nurses completed at least some part of the survey. We eliminated data from those nurses who did not complete at least 90% of the measures central to the current study. To control for careless responding, we also eliminated from further consideration data from those nurses who spent less than 600 s (10 min) or more than 10,000 s (166 min) on the survey. We reasoned that these time limits represented a conservative control given that we estimated a generous 30 min completion time for the survey in pilot testing. We focused on nurses working in the same political and geographical location to control for the wide array of circumstances that might have affected nurses’ experiences based on geographical location, and thus we eliminated from further consideration those nurses who did not work in Texas. The final sample included N = 367 nurses (*n* = 309 women). The sample was relatively educated: 56% held a Bachelor’s degree, 32% a Master’s degree, 8% had an associate’s degree, and 5% had Ph.D.s in nursing. As we were working with a convenience sample, we did not conduct a prior power analysis. However, a post-hoc analysis using G-power showed that a sample of 367 nurses results in power of 0.97 (α = 0.05, two tailed) to find a small to moderate sized correlation (*r* = 0.20) [58].

**Figure 1:**
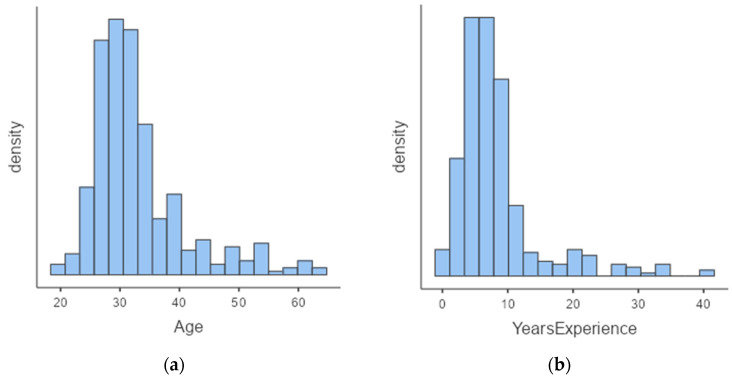
Panel (**a**) is a histogram of the age of the sample; Panel (**b**) is a histogram of the sample’s years of experience. N = 376.

The average age of nurses in our sample was 33.2 years (SD = 8.01), with a range of 20 to 64 years. The average years of experience of the nurses in our study was 8.25 years (SD = 6.44), with a range of 0.5 to 40 years. The distribution of age and experience show positive skews (Figure 1; Panel A for age and Panel B for experience). We removed outliers (+/− 3 SD above and below the mean), which resulted in eliminating older nurses, and the pattern of relationships among these variables remained largely unchanged (a correlation table of these relationships is available in Appendix A, Table A1). Because of the similarity of results with and without outliers, and because we wanted to retain older nurses in our sample, we chose to leave outliers in our sample. We acknowledge the relative youth of our sample, but note that the age of our sample is similar to other research on age, burnout, and coping (e.g., a mean age of 35) reported in [35]. Further, meta-analytic research on burnout discussed below reports similar age ranges [3].

### 2.2. Measures

Demographic information: Nurses provided their age (in years), gender, and responded to the question, “How many years have you been a nurse?”. Nurse age and experience were analyzed as continuous variables.

Coping: We used Carver’s 28-item Brief COPE assessment to measure positive and negative aspects of coping [36]. Nurses were instructed to consider whether they had been using various ways of coping over the previous two weeks, regardless of whether the coping strategy seemed to be working or not. Nurses responded on a four-point scale where *1 = I haven’t been doing this at all* and *4 = I’ve been doing this a lot.* All participants responded to items in the same order; however, items were randomly sorted such that they were not grouped by content. The Brief COPE assessment is designed to assess 14 facets of coping with two-items each. However, we did not analyze the 14 coping facets for two reasons. First, internal consistency reliability estimates for these 14 facets were low in our sample, with only two of the fourteen scales exceeding reliability estimates over α = 0.70 (the Cronbach’s internal consistency reliability estimates ranged from α = 0.34 to 0.74 for the two-item scales [59]). Second, and more importantly, we did not have research questions about specific coping facets. Rather, we were interested in the effect of generally positive and negative coping strategies on burnout for nurses. We thus conducted an exploratory factor analysis using maximum likelihood and varimax rotation, specifying the extraction of two factors for positive and negative coping, which accounted for 26% of the variance. The EFA resulted in a 14-item positive coping strategy scale (e.g., “I’ve been taking action to try to make the situation better”, and “I’ve been looking for something good in what is happening”; α = 0.79; McDonald’s ω = 0.79) and a 10-item negative coping strategy scale (e.g., “I’ve been blaming myself for things that happened”, and “I’ve been using alcohol or other drugs to make myself feel better”; α = 0.85; McDonald’s ω = 0.79). The four items that did not clearly load on the positive or negative coping strategy facets were not used in this analysis and tended to reference using jokes, expressing negative emotions, and using distracting strategies, such as watching movies/TV. The full factor analysis, with items and factor loadings, is in Appendix B (Table A2). Additionally included in Appendix B is the mean and standard deviation associated with each item, which provides information about the most highly endorsed coping approaches reported by our sample. The coping items that loaded on the positive coping factor tended to represent approach-based coping (e.g., “I’ve been thinking hard about what steps to take”.) and the items that loaded on negative coping tended to represent avoidance-based coping (e.g., I’ve been refusing to believe that it has happen”.). However, the factors also include items that describe other types of behaviors that represent positive (e.g., I’ve been learning to live with it) and negative (e.g., “I’ve been saying things to let my unpleasant feelings escape”), thus we call these factors positive and negative coping rather than approach and avoidance coping.

Burnout. We used Maslach and Jackson’s scale, which assesses burnout along three dimensions: emotional exhaustion, depersonalization, and personal accomplishment [2]. Nurses were asked to consider their agreement with a set of statements and asked to provide their response within the context of the pandemic (i.e., “since the outbreak of COVID-19”). All items were assessed on a five-point Likert scale from *1 = strongly disagree* to *5 = strongly agree*. Note that the response scale we used differed from that recommended by Maslach and Jackson, which asks about frequency of thoughts related to burnout experienced from “a few times a year” to “everyday.” Because we wanted to understand nurses’ experiences since the outbreak of COVID-19 in Texas (i.e., since March 2020), which was anywhere from 2 to 4 months from the time nurses took the survey (i.e., May to July 2020), response options that included “a few times a year” no longer made sense, so we changed the response scale to agree/disagree. Emotional exhaustion was assessed with seven items (e.g., “I feel emotionally drained from my work”, and “I feel used up at the end of the workday”; α = 0.85); personal accomplishment was assessed with eight items (e.g., “I can easily understand how my recipients feel about things”, and “I deal very effectively with the problems of my recipients”; α = 0.78); and depersonalization was assessed with five items (e.g., “I feel I treat some recipients as if they were impersonal ‘objects’”, and “I worry that this job is hardening me emotionally”; α = 0.80).

### 2.3. Data Analysis

To understand our sample of nurses working in Texas during the COVID-19 pandemic relative to other healthcare workers during this same period, we first compared levels of burnout in our sample with those of a meta-analysis of burnout in healthcare workers during COVID-19 [3]. We chose to compare with a meta-analysis because it is a compilation of multiple studies carried out roughly during the same period as our data collection. As did the meta-analysis, we categorized nurse burnout into low, moderate, and severe groups using the following cut scores: for emotional exhaustion, low ≤16, moderate ≥17 and ≤26, and severe was ≥27; for depersonalization, low ≤6, moderate was ≥7 and ≤12, and severe was ≥13. For personal accomplishment, low ≤20, moderate was ≥21 and ≤37, and high was ≥38 [3]. For all other analyses, we used a correlation/regression approach to data analysis given the continuous nature of our predictors (age/experience), mediators (coping), and outcome (burnout) measures. We used path analysis, particularly related to direct and indirect effects, to examine mediating effects. All data cleaning and analyses: descriptive, reliability analyses, correlational, regression, and path analyses were conducting in Jamovi version 2.2.5 [60].

## 3. Results

Table 1 shows results of the comparison of levels of burnout experienced by our sample compared to meta-analytic results from the Parandeh et al. meta-analysis [3]. Although there are differences in percentages for severe, moderate, and low categories of burnout, combining moderate and severe levels of burnout reveals similar levels as the meta-analysis. Specifically, we found that 81.6% of nurses in our sample reported moderate to severe levels of emotional exhaustion; 92.3% reported moderate to severe levels of depersonalization. We also found that 97.3% of nurses reported low to moderate personal accomplishment. Notably, the age of healthcare workers during the pandemic included in the meta-analysis ranged from 30.9 to 46.4 years [3], similar to our relatively young sample.

Descriptive statistics, reliability estimates, and inter-correlations among study variables are shown in Table 2. As can be seen in the table, age and job experience were highly correlated *(r* = 0.85, *p* < 0.001). Hypothesis 1 stated that age (H1a) and job experience (H1b) would be negatively correlated with burnout and this hypothesis received mixed support. Age was significantly positively related to personal accomplishment (*r* = 0.22, *p* < 0.001) as was job experience (*r* = 0.13, *p* = 0.017). Age and years of experience were also significantly negatively related to depersonalization (*r* = −0.34, *p* < 0.001 for age and *r* = −0.30, *p* < 0.001 for years of experience) as expected. Neither age nor experience were significantly related to emotional exhaustion.

Hypothesis 2 stated that positive coping would be positively related to personal accomplishment (2a) but negatively related to emotional exhaustion and depersonalization (2b and 2c respectively). We also found mixed support for this hypothesis. Specifically, we found that positive coping was positively related to personal accomplishment (*r* = 0.44, *p* < 0.001). However, positive coping was positively related to emotional exhaustion (*r* = 0.18, *p* < 0.001) and was not significantly related to depersonalization. Similarly, Hypothesis 3 stated that negative coping would be negatively related to personal accomplishment (3a) but positively related to emotional exhaustion (3b) and depersonalization (3c). This hypothesis was supported as negative coping was significantly negatively related to personal accomplishment (*r* = −0.17, *p* < 0.001) and positively related to emotional exhaustion (*r* = 0.32, *p* < 0.001) and depersonalization (*r* = 0.58, *p* < 0.001).

Hypotheses 4 stated that age and job experience would be positively related to using positive coping strategies and negatively related to using negative coping strategies. This hypothesis also garnered mixed support. Age was significantly positively related to using positive coping strategies (H4a), although this effect was small (*r* = 0.11, *p* = 0.027), but job experience was not significantly correlated with positive coping, finding no support for H4b. As predicted, age (H5a) and job experience (H5b) were significantly negatively related to using negative coping strategies, and these effects were medium in magnitude (i.e., *r* = −0.32, *p* < 0.001 for age and negative coping and *r* = −0.31, *p* < 0.001, for job experience and negative coping [61].

**Figure 2:**
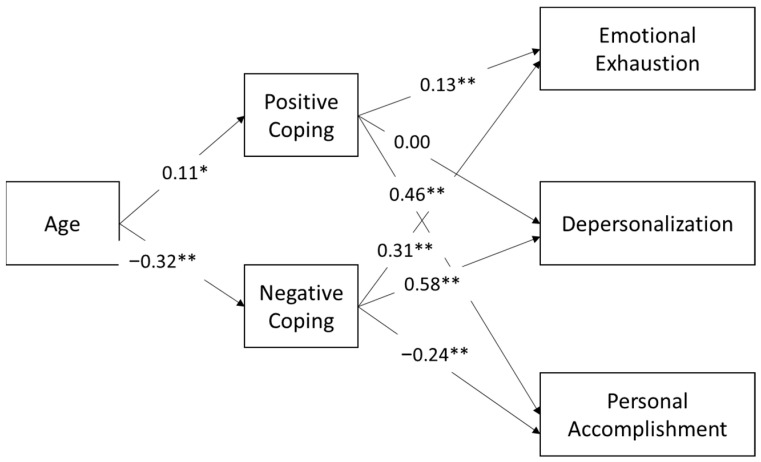
Path analysis of the relationship between age and burnout as mediated by positive and negative coping. * *p* < 0.05, ** *p* < 0.01.

We used path analysis to test Hypothesis 6, that coping strategies would mediate the relationship between age and job experience and burnout. To understand the effects of age and job experience separately, we first ran a model using age as the exogenous variable, positive and negative coping are the mediating variables, and the three facets of burnout are the dependent variables. Parameter estimates are shown in Figure 2 and the upper portion of Table 3; indirect effects are shown on the bottom of Table 3. Age was significantly positively associated with positive coping (β = 0.114, *p* = 0.026) and negatively associated with negative coping strategies (β = −0.321, *p* < 0.001). Both positive and negative coping were significantly related to all facets of burnout with the exception of a non-significant relationship between positive coping strategies and depersonalization. A test of indirect effects in the model determined that the effect of age on personal accomplishment was mediated by positive coping strategies (β = 0.053, *p* = 0.029), the effect of age on emotional exhaustion was mediated by negative coping strategies (β = −0.098, *p* < 0.001), as was the effect of age on depersonalization (β = −0.186, *p* < 0.001). The effect of age on personal accomplishment was also mediated by negative coping strategies (β = 0.078, *p* < 0.001). We also found evidence for a significant mediating effect of positive coping strategies on the relationship between age and emotional exhaustion at the *p* < 0.10 level (β = 0.015, *p* = 0.089).

**Figure 3:**
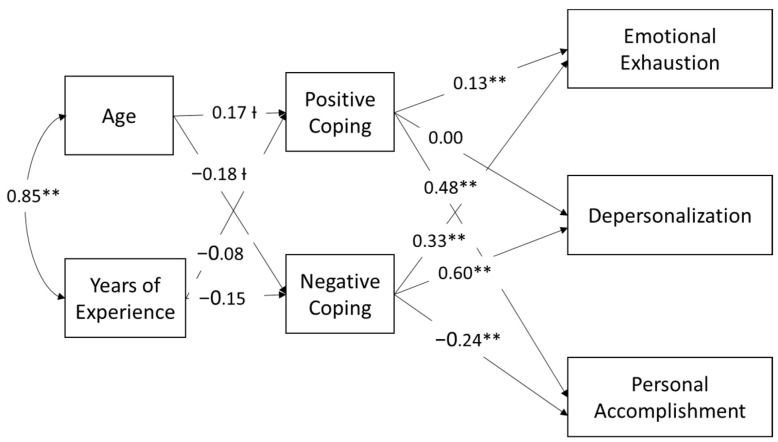
Path analysis of the relationship between age, job experience, and burnout as mediated by positive and negative coping. Ɨ *p* < 0.10, ** *p* < 0.01.

We included years of experience in a second model to examine the effects of age and job experience estimated simultaneously. Parameter estimates are shown in Figure 3 and on the top portion of Table 4. Including nurse experience reduced the effect of age on both positive and negative coping to be significant at the *p* < 0.10 level: β = 0.17, *p* = 0.085 for positive and β = −0.18, *p* = 0.051 for negative coping. However, years of experience was not significantly associated with either positive or negative coping in this model. The patterns of relationships between coping and burnout in the model including job experience were the same as those in the model including age only. The indirect effects of age on burnout also attenuated in this model when years of experience was accounted for; some of these relationships were rendered no longer significant and others significant now at the *p* < 0.10 level (indirect effects are shown at the bottom of Table 4). At the *p* < 0.10 level, the effect of age on personal accomplishment was mediated by positive coping strategies (β = 0.081, *p* = 0.089), the effect of age on emotional exhaustion was mediated by negative coping strategies (β = −0.062, *p* = 0.060), as was the effect of age on depersonalization (β = −0.111, *p* = 0.053). The effect of age on personal accomplishment was also mediated by negative coping strategies (β = 0.044, *p* = 0.066). We also found that the indirect effect of positive coping strategies on the relationship between age and emotional exhaustion was no longer significant. There were no significant indirect effects of coping on the relationship between years of experience and burnout (as shown in the bottom portion of Table 4). In sum, analyzing age alone provides some evidence for the mediating effect of coping on the age-burnout relationship (H6), in particular as related to positive coping strategies mediating the effect of age on personal accomplishment and the effect of age on emotional exhaustion and depersonalization being mediated by negative coping strategies, but these indirect effects were no longer significant when experience was also included in the model.

## 4. Discussion

We used a lifespan development approach to frame hypotheses about the benefits of age and job experience for reducing burnout via coping strategies. Specifically, we hypothesized that age and experience would be related to practicing more positive, and fewer negative, coping strategies, which would reduce burnout. Data were collected during the first wave of the COVID-19 pandemic in a cross-sectional study using a convenience sample of 376 nurses working in acute care hospitals in Texas. A comparison of our sample of nurses with meta-analytic research on burnout experienced by healthcare professionals during the pandemic suggests that our sample is similar in terms of level of burnout and age [3]. We expected that age and job experience would be negatively related to burnout in the face of COVID-19 and that older and more experienced nurses would have better coping strategies, leading to reduced burnout. As predicted, we found that age was positively associated with positive coping and negatively associated with negative coping strategies. Similarly, nurses who reported being on the job longer were likely to report using fewer negative coping strategies, but job experience was not significantly related to positive coping strategies. We also found that negative coping strategies were significantly related to burnout as expected. Specifically, negative coping approaches were negatively related to feelings of personal accomplishment, but positively related to emotional exhaustion and depersonalization. Our results concerning positive coping strategies and burnout were more mixed. Specifically, more positive coping was associated with feelings of personal accomplishment, but were also associated with emotional exhaustion, and not related to depersonalization. It could be that those reporting engaging in more coping strategies—whether positive or negative—experienced more stress that necessitated the use of these strategies, which led to emotional exhaustion.

To understand the extent to which coping mediates the relationship between age and burnout, we first modeled the data with age and then with age and job experience together. In the model with only age, older nurses’ negative relationship with burnout was associated with their avoiding negative coping strategies. Although we did not find a main effect of age on emotional exhaustion, when all variables are estimated simultaneously in the path analysis, we did find a significant indirect effect of age on emotional exhaustion through negative coping. This finding suggests that older nurses are less likely to engage in negative coping behavior, which is related to a reduction in all facets of burnout, including emotional exhaustion. These finding align with research on coping and stress conducted during the pandemic. Specifically, a study of medical staff working in Italy during the pandemic suggests that older healthcare workers engaged in less negative coping. This study also found that age was negatively related to perceived secondary trauma and that coping was an important mediator between perceived emergency stress and secondary trauma. This study, did not, however, examine whether coping mediated the negative relationship between age and secondary trauma [45].

We also found that positive coping mediated the relationship between age and personal accomplishment, which suggests that age is related to more approach/adaptive coping, which will have an effect on a nurse’s sense of efficacy on the job. This finding is also aligned with prior research examining the use of mature coping strategies (i.e., affiliation, altruism, and anticipation) compared to neurotic (e.g., suppression, dissociation) and immature (e.g., denial, and acting out) coping strategies with healthcare workers during COVID-19. This study found that mature coping was positively related to personal accomplishment and resilience and that immature and neurotic coping was related to perceived stress and burnout. This study also reported that older healthcare workers experienced less stress and burnout but did not examine whether coping mediated the relationship between age and these outcomes. Thus, one contribution of the current study is that it suggests that age can be a protective factor in that it leads to more positive and less negative coping, which in turn affects the experience of burnout.

Further, our results suggest age is more consistently related to avoiding negative coping than engaging in positive coping behavior. It is perhaps somewhat surprising that it is the *lack* of engaging in negative coping behaviors, rather than engaging in positive coping, that seems to be most important for older nurses. Upon further reflection, however, we think these effects are likely a function of the extreme environment in which nurses operated at the time of our study. That is, engaging in positive coping strategies may take more time, attention, and effort (e.g., getting advice from others; thinking about a strategy to improve one’s situation) compared to a more passive approach, such as avoiding negative behaviors. Avoiding negative coping may be akin to focusing on positive versus negative stimuli, or the positivity effect as suggested by lifespan development theories [25]. Similar to other studies, our findings suggest that more passive approaches may be most effective. For example, a study of healthcare professionals during the pandemic reported that a positive attitude about the situation (perhaps a more passive coping approach) was more protective against burnout than more active strategies like seeking social support [42]. Future research could be conducted to examine effort associated with an array of coping mechanisms.

It is also important to ask what our results say about the role of job experience relative to age. Because age and job experience are highly correlated in our sample, it is not surprising that the relationships between age and the mediating variables were reduced when job experience was added to the model. Moreover, the high correlation makes it difficult to examine age and experience independently. Nonetheless, within a lifespan development framework, we consider both age and job experience to be proxies for the experience acquired through one’s job and more generally in life [10]. An important and interesting future research project aligned with lifespan development theory would be to understand the types of life experiences that generally support productive coping strategies that generalize across work and more general life domains.

### 4.1. Theoretical Implications

The current study adds to the lifespan development literature by highlighting an advantage associated with age and experience: the ability to deal with stressful environments and experience relatively less burnout, and a tendency to engage in positive (and to avoid negative) coping strategies. Notably, most of the lifespan development literature speaks to the advantages of emotion regulation acquired with age in the context of daily interactions (e.g., everyday problem solving [11]). By contrast, the current study examines—and finds—age advantages in an extreme and highly stressful work environment, suggesting that the age and experience advantages associated with emotion regulation are robust. One consideration, however, is that the sample of nurses in the current study was relatively young. Although not examining performance in extreme environments, Reh et al. did not find an advantage of experience for mitigating the effects of job demands on emotional well-being and job satisfaction, perhaps because their sample was significantly older [12]. Future research can further examine the boundary conditions associated with increased emotion regulation with age, but will need to consider the vast variability in the growth and decline of abilities with age [24].

### 4.2. Practical Implications

Burnout is a significant emotional and physical fatigue that results in the loss of motivation and can evolve into total exhaustion and is a significant factor in leaving the workforce [2]. The supply of nurses decreased by more than 100,000 from 2020 to 2021 [62]. A substantial number of nurses leaving the workforce are under the age of 35, and most are employed in hospitals [62], and thus it is imperative to understand the factors that lead to—or that can mitigate—burnout.

More experienced nurses may develop positive coping skills over the years that can help them to reduce burnout and maintain a sense of well-being. It would be interesting and informative to understand what these coping strategies are. In an integrative literature review, de Oliveria and colleagues identify how coping skills training, resilience skills training, meditation to reduce stress, and yoga are strategies reported to reduce facets of burnout [63]. They also identified mental health programs, including audio-video recorded mental exercises, psychological trainings, and professional identity development programs. Physical activity incentives, Reiki, Touch of Healing, Therapeutic Massage, Jin Shin, Jyutsu, Guided Imagery, centering actions on the meaning of job satisfaction and quality of life, and changes in the environment may also lead to decreased burnout [63]. Spiritual practices can provide hope and inspiration in difficult situations [46]. By incorporating these coping skills into their daily routines, nurses can effectively manage burnout and maintain a sense of well-being in their work.

These strategies may be effective, but they also require extra effort and time to incorporate into a daily routine. Our findings suggest that in extreme environments—when extra time and effort are not feasible—effective coping behavior is perhaps more about what a person does not do than what they do. That is, the avoidance of negative coping strategies (e.g., negative self-talk, avoiding substance use) can be effective at mitigating the effects of burnout. Although we cannot speak to the exact strategies used by nurses in our sample, we do know that older and more experienced nurses tended to avoid negative strategies. Future research can focus on older/more experienced nurses’ experiences and how they prevent burnout and how they may mentor junior nurses through mentorship programs that foster resilience to prevent burnout. Active involvement in burnout prevention or intervention using personal or organizational intervention and social support can improve symptoms of burnout [63].

### 4.3. Limitations

As does every study, the current study has limitations. First, it is cross sectional, and thus we are unable to disentangle causal relationships. Although we model mediation, we acknowledge that these models are only suggestive given that all data were collected at the same time and we cannot isolate method variance. Moreover, because we did not collect data on pre-pandemic levels of burnout or on prior levels of emotional stability, we cannot know the extent to which prior levels of these variables are proxies for the age and experience markers used in this study. Ideally, we would have been able to conduct a longitudinal study that first assessed baseline levels of burnout and psychological factors pre-pandemic, then assessed coping strategies at a mid-pandemic time point, and finally assessed burnout levels. Ideally the coping and burnout assessments would have been separated by enough time to limit method-related variance but would capture the same pandemic intensity. However, capturing data from nurses during a healthcare crisis that was impossible to predict made such an approach impossible. Our design is limited, but our results are suggestive that age/experience and coping strategies are related to nurse burnout. Future longitudinal research in a more controlled environment would be necessary to establish causal relationships.

Collecting data in one state in the U.S. (Texas) may also limit the generalizability of our findings. The decision to limit data collection to one state was a purposeful one at the time of data collection given that every state in the U.S. had different experiences with the COVID-19 virus and political and other factors related to public health and funding. Thus, we consider the relatively limited scope of our sample an advantage. That is, we can assume, given what we know about conditions in Texas at the time of data collection (May–July 2020), nurses were experiencing extreme conditions.

We also collected data with a convenience sample of nurses. That is, we took the opportunity to examine nurses’ experiences with coping and burnout during the pandemic and did not have enough information about this sample to anticipate how age and experience would be distributed therein. As such, our sample is relatively range restricted in terms of age and experience. Even with this range restriction, however, we find significant effects of age and experience, particularly as related to coping and burnout. It would be interesting to examine these factors in a more carefully selected sample (i.e., a sample stratified on age and job experience), which might provide information about the age and experience levels at which nurses are most likely to engage in productive coping strategies and experience less burnout.

An additional limitation associated with the sample is related to a survival effect. That is, we are positing that reduced burnout and better coping are a function of age and experience within a lifespan development perspective. One alternative hypothesis is that only nurses who have better coping skills and who experience less burnout *stay* in the job of nursing. Indeed, survival within a job role is an issue related to any study of workplace aging and must be considered when interpreting the results [64]. A long-term longitudinal study would be necessary to disentangle these effects. Such a study is recommended for future research.

Another limitation of the current study is associated with the measurement of coping. As described above, we reduced the 14 facets of the Brief COPE through EFA to obtain more general factors for positive and negative coping and we were unable to validate this factor structure with an external sample. We acknowledge that it may have been better to use a coping inventory focused more generally on positive and negative coping in this study. However, we reasoned that the Brief COPE was designed to be flexible [36], and the factor structure had been reduced through EFA in previous research [20,65]. Although the measurement of coping is perhaps not ideal in the current study, our results show many of the expected relationships with burnout and some of the expected relationships with age and experience. Relatedly, we did not ask nurses to explicitly describe how they were coping with the pandemic (i.e., through an open-ended questionnaire). This approach may have provided insight into productive and unproductive coping and age/experiences advantages. Furthermore, we are not able to link specific coping strategies to different types of burnout, nor do we examine external factors that might mitigate or exacerbate burnout like living conditions, family, and socio-economic status (wealth). Questions related to other person-variables related to burnout, and how specific coping strategies are related to burnout, are promising areas of future research.

## 5. Conclusions

The finding that age and experience were negatively related to burnout and associated with productive coping strategies in an extreme environment is an important extension of lifespan development models and provides evidence in support of the benefits of aging. Although it is important to understand how burnout manifests in extreme environments, prevention of burnout is also an important consideration. Our results further suggest that younger workers may be able to learn from their more experienced and older colleagues to engage in more productive coping strategies in extreme, and perhaps also day-to-day, work environments.

## Figures and Tables

**Table 1:** Nurses falling within severe, moderate, and low levels of burnout for the current study compared with a meta-analysis of burnout in healthcare workers during the pandemic [3].

	Emotional Exhaustion		Depersonalization		Personal Accomplishment	
Level	Current Study	Parandeh et al. [3]	Current Study	Parandeh et al. [3]	Current Study	Parandeh et al. [3]
Severe/high	99 (26.3%)	37% [27–48%]	280 (74.5%)	18% [10–25%]	10 (2.7%)	
Moderate	208 (55.3%)	45% [22–68%]	67 (17.8%)	49% [10–89%]	358 (95.2%)	38% [12–65%]
Low	69 (18.4%)		29 (7.7%)		8 (2.1%)	51% [25–77%]

Note: N = 376 for the current study. Moderate and severe emotional exhaustion were scores ≥17 and ≥27, respectively; moderate and severe depersonalization were scores ≥7 and ≥13; and moderate and severe reduced personal accomplishment were ≤38 and ≤21 on the Maslach and Jackson burnout scale [2]. Brackets represent 95% confidence intervals as reported in Parandeh et al. [3]. Low levels of emotional exhaustion and depersonalization and high levels of personal accomplishment were not reported by Parandeh et al.

**Table 2:** Means, standard deviations, inter-correlations, and internal consistency reliability estimates for study variables. (N—376).

		M	SD	1	2	3	4	5	6	7
1	Age	33.2	8.01	—						
2	Years of Experience	8.25	6.44	0.85 ***	—					
3	Positive Coping Strategies	2.81	0.46	0.11 *	0.07	(0.79)				
4	Negative Coping Strategies	2.25	0.67	−0.32 ***	−0.31 ***	0.16 **	(0.85)			
5	Personal Accomplishment	3.71	0.61	0.22 ***	0.13 *	0.44 ***	−0.17 ***	(0.78)		
6	Emotional Exhaustion	3.19	0.88	−0.04	0.01	0.18 ***	0.32 ***	−0.05	(0.85)	
7	Depersonalization	3.04	0.96	−0.34 ***	−0.30 ***	0.09	0.58 ***	0.03	0.31 ***	(0.80)

Note. * *p* < 0.01, ** *p* < 0.01, *** *p* < 0.001. Internal consistency reliability estimates (Cronbach’s alpha) are shown on the diagonal.

**Table 3:** Parameter estimates for mediation model with age as the exogenous variable, coping as mediating variables, and burnout as the outcome variable: Direct effects are shown in the top part of the table; indirect effects are shown in the bottom of the table.

				95% CI			
Endogenous	Exogenous	Estimate	SE	Lower	Upper	β	*p*
Positive Coping	Age	0.006	0.003	7.96 × 10^−4^	0.012	0.114	0.026
Negative Coping	Age	−0.027	0.004	−0.035	−0.019	−0.321	<0.001
Emotional Exhaustion	Positive Coping	0.241	0.092	0.061	0.422	0.128	0.009
Emotional Exhaustion	Negative Coping	0.399	0.064	0.274	0.524	0.305	<0.001
Depersonalization	Positive Coping	−0.009	0.088	−0.181	0.163	−0.004	0.917
Depersonalization	Negative Coping	0.840	0.061	0.721	0.959	0.58	<0.001
Personal Accomplishment	Positive Coping	0.625	0.059	0.51	0.74	0.465	<0.001
Personal Accomplishment	Negative Coping	−0.226	0.041	−0.306	−0.147	−0.243	<0.001
				**95% CI**			
**Indirect Effects**		**Estimate**	**SE**	**Lower**	**Upper**	**β**	***p***
Age ⇒ Positive Coping ⇒ Emotional Exhaustion		0.002	0.001	0.000	0.003	0.015	0.089
Age ⇒ Positive Coping ⇒ Depersonalization		0	0.001	−0.001	0.001	−0.001	0.917
Age ⇒ Positive Coping ⇒ Personal Accomplishment		0.004	0.002	0.000	0.008	0.053	0.029
Age ⇒ Negative Coping ⇒ Emotional Exhaustion		−0.011	0.002	−0.015	−0.006	−0.098	<0.001
Age ⇒ Negative Coping ⇒ Depersonalization		−0.022	0.004	−0.03	−0.015	−0.186	<0.001
Age ⇒ Negative Coping ⇒ Personal Accomplishment		0.006	0.001	0.003	0.009	0.078	<0.001

Note: Direct effects (i.e., path coefficients) are also shown in Figure 2. Emotional exhaustion, depersonalization, and personal accomplishment are indicators of burnout.

**Table 4:** Parameter estimates for mediation model with age and job experience as the exogenous variables, coping as mediating variables, and burnout as the outcome variable: Direct and indirect effects.

				95% CI			
Endogenous	Exogenous	Estimate	SE	Lower	Upper	β	*p*
Positive Coping	Age	0.01	0.006	−0.001	0.022	0.171	0.085
Positive Coping	Years of Experience	−0.005	0.007	−0.019	0.008	−0.076	0.441
Negative Coping	Age	−0.016	0.008	−0.031	0.000	−0.184	0.051
Negative Coping	Years of Experience	−0.016	0.01	−0.034	0.003	−0.152	0.107
Emotional Exhaustion	Positive Coping	0.248	0.092	0.068	0.428	0.132	0.007
Emotional Exhaustion	Negative Coping	0.441	0.065	0.314	0.568	0.334	<0.001
Personal Accomplishment	Positive Coping	0.635	0.059	0.52	0.751	0.476	<0.001
Personal Accomplishment	Negative Coping	−0.226	0.042	−0.307	−0.144	−0.24	<0.001
Depersonalization	Positive Coping	0.001	0.086	−0.167	0.170	6.47 × 10^−4^	0.988
Depersonalization	Negative Coping	0.863	0.06	0.744	0.981	0.60	<0.001
			**95% CI**				
**Indirect Effects**	**Estimate**	**SE**	**Lower**	**Upper**	**β**	***p***	
Age ⇒ Positive Coping ⇒ Emotional Exhaustion	0.003	0.002	−0.001	0.006	0.023	0.147	
Age ⇒ Positive Coping ⇒ Personal Accomplishment	0.006	0.004	−0.001	0.014	0.081	0.089	
Age ⇒ Positive Coping ⇒ Depersonalization	0.000	0.001	−0.002	0.002	0.000	0.988	
Age ⇒ Negative Coping ⇒ Emotional Exhaustion	−0.007	0.004	−0.014	0.000	−0.062	0.06	
Age ⇒ Negative Coping ⇒ Personal Accomplishment	0.004	0.002	0.000	0.007	0.044	0.066	
Age ⇒ Negative Coping ⇒ Depersonalization	−0.013	0.007	−0.027	0.000	−0.111	0.053	
Experience ⇒ Positive Coping ⇒ Emotional Exhaustion	−0.001	0.002	−0.005	0.002	−0.010	0.459	
Experience ⇒ Positive Coping ⇒ Personal Accomplishment	−0.003	0.005	−0.012	0.005	−0.036	0.443	
Experience ⇒ Positive Coping ⇒ Depersonalization	0.000	0.000	−0.001	0.001	0.000	0.988	
Experience ⇒ Negative Coping ⇒ Emotional Exhaustion	−0.007	0.004	−0.015	0.002	−0.051	0.117	
Experience ⇒ Negative Coping ⇒ Personal Accomplishment	0.004	0.002	−0.001	0.008	0.037	0.122	
Experience ⇒ Negative Coping ⇒ Depersonalization	−0.013	0.008	−0.03	0.003	−0.091	0.109	

Note: Emotional exhaustion, depersonalization, and personal accomplishment are indicators of burnout.

## Data Availability

The data presented in this study are available on request from the corresponding author. The data are not publicly available due to privacy concerns.

## Appendix A

**Table A1:** SD’s above and below the mean. N = 346.

	1	2	3	4	5	6
1. Age	—					
2. Years of Experience	0.792 **	—				
3. Positive Coping	0.082	0.069	—			
4. Negative Coping	−0.183 **	−0.172 **	0.198 **	—		
5. Personal Accomplishment	0.208 **	0.126 *	0.445 **	−0.16 **	—	
6. Emotional Exhaustion	0.025	0.026	0.214 **	0.372 **	−0.028	—
7. Depersonalization	−0.206 **	−0.178 **	0.143 **	0.569 **	0.075	0.314 **

Note. ** p* < 0.05; *** p* < 0.01.

## Appendix B

**Table A2:** Factor Analysis of the Brief COPE Inventory (N = 376).

	Factor			
Item	Negative Coping	Positive Coping	Uniqueness	M (SD)
I’ve been blaming myself for things that happened.	0.717		0.483	2.23 (1.02)
I’ve been refusing to believe that it has happen.	0.675		0.536	2.18 (1.03)
I’ve been saying to myself “this isn’t real”.	0.650		0.575	2.27 (1.02)
I’ve been using alcohol or other drugs to help me get through it.	0.631		0.598	2.09 (1.00)
I’ve been criticizing myself.	0.612		0.613	2.31 (1.00)
I’ve been giving up trying to deal with it.	0.594		0.623	2.21 (1.06)
I’ve been using alcohol or other drugs to make myself feel better.	0.580		0.659	2.14 (1.03)
I’ve been saying things to let my unpleasant feeling escape.	0.569		0.645	2.36 (0.94)
I’ve been giving up the attempt to cope.	0.562		0.682	2.43 (1.04)
I’ve been making fun of the situation.	0.446		0.800	2.29 (1.01)
I’ve been taking action to try to make the situation better.		0.581	0.654	2.89 (0.89)
I’ve been thinking hard about what steps to take.		0.554	0.688	2.85 (0.84)
I’ve been looking for something good in what is happening.		0.504	0.745	2.87 (0.87)
I’ve been trying to see it in a different light, to make it seem more positive.		0.482	0.765	2.80 (0.88)
I’ve been trying to get advice or help from other people about what to do.		0.480	0.751	2.69 (0.86)
I’ve been concentrating my efforts on doing something about the situation I’m in.		0.473	0.768	2.81 (0.91)
I’ve been trying to come up with a strategy about what to do.		0.473	0.772	2.84 (0.87)
I’ve been learning to live with it.		0.457	0.781	2.97 (0.80)
I’ve been getting comfort and understanding from someone.		0.446	0.800	2.84 (0.90)
I’ve been trying to find comfort in my religion or spiritual beliefs.		0.441	0.795	2.65 (0.97)
I’ve been getting emotional support from others.		0.395	0.816	2.81 (0.86)
I’ve been praying or meditating.		0.391	0.842	2.60 (1.02)
I’ve been getting help and advice from other people.		0.374	0.838	2.78 (0.91)
I’ve been accepting the reality of the fact that it has happened.		0.346	0.872	2.97 (0.90)
I’ve been turning to work or other activities to take my mind off things.			0.893	2.62 (0.91)
I’ve been doing something to think about it less, such as going to the movies, watching TV, reading, daydreaming, sleeping, or shopping.			0.929	2.58 (0.90)
I’ve been expressing my negative feelings.			0.886	2.43 (0.92)
I’ve been making jokes about it.			0.906	2.36 (0.99)

Note: Exploratory factor analysis using maximum likelihood and varimax rotation, specifying the extraction of two factors for positive and negative coping. M = mean and SD = standard deviation on a 4-point scale where *1 = I haven’t been doing this at all* and *4 = I’ve been doing this a lot.* Items available on page 96 of [36].
